# Reproductive health literacy scale: a tool to measure the effectiveness of health literacy training

**DOI:** 10.1186/s12978-025-01959-6

**Published:** 2025-01-30

**Authors:** Maha Rauf, Zahra Goliaei, Lana Machta, Jenny Chang, Heike Thiel de Bocanegra

**Affiliations:** 1 Department of Obstetrics and Gynecology, School of Medicine, University of California Irvine, Irvine, CA USA; 2 Public Health Program, College of Education and Health Science, Touro University of California, Vallejo, CA USA; 3 Department of Medicine, University of California Irvine, Irvine, CA USA

**Keywords:** Reproductive health, Health literacy, Refugee, Digital health literacy, Scale development

## Abstract

**Background:**

Refugee women’s reproductive health (RH) outcomes have been impacted by several factors, including experiencing war, lack of access to healthcare, and possible gender-based violence. After resettlement, low health literacy, financial difficulties, cultural and linguistic barriers, and unfamiliarity with the healthcare system also add to the preexisting barriers. Although several efforts have focused on health education and improving health literacy among refugee women, there has not been a validated tool to measure the effectiveness of these trainings and their possible impact. This study aims to adapt a culturally and linguistically appropriate survey that helps address this gap.

**Methods:**

We conducted a literature review to identify the existing tools and identified possible domains and items supporting RH literacy measures. The identified items were collected and adapted as a single scale with three domains: (a) general health literacy, measured with HLS-EU-Q6, (b) digital health literacy, measured with eHEALS, and (c) reproductive health literacy, measured through a composite of the Cervical Cancer Literacy Assessment Tool (C-CLAT) and the Refugee Reproductive Health Network (ReproNet) postpartum literacy scale. After content validity and face validity of the adapted scale, it was translated into Dari, Arabic, and Pashto and was administered to participants of RH literacy training sessions.

**Results:**

A total of 67 Dari, 53 Arabic, and 64 Pashto-speaking refugee women completed the survey. The mean scores obtained between the three language groups were similar in the domains of digital health literacy and reproductive health literacy (*p* > 0.05), whereas the scores for general health literacy were not (*p* > 0.05). The inter-item reliability score for the domains of general health literacy, digital health literacy and RH literacy across all three language groups was above *α* = 0.7.

**Conclusion:**

This scale addresses the need for validated tools to measure reproductive health literacy. It has the promise to provide a tool for assessing the effectiveness of health interventions on health literacy. Future applications can utilize this scale to investigate the differences in health literacy in refugee populations speaking Dari, Pashto, and Arabic.

## Background

Since 2008, 963,748 refugees who have been forced to migrate from their countries of origin due to political instability, persecution, conflict, violence, or human rights violations have resettled in the United States [1]. 131,424 Special Immigrant (SIV) Visas have been granted to Afghan and Iraqi nationals since 2014 [2]. In August 2021, Sacramento County emerged as one of the leading destinations for Afghan refugees, welcoming over 16,525 individuals, including refugees and SIV holders since then [2].

Forced immigration is a known risk factor for decreasing access and utilization of sexual and reproductive health (SRH) services [3, 4]. The refugee experience may leave a lasting impact on individuals’ health, rendering them especially vulnerable to poor health outcomes [5]. On a global scale, women who were forced to migrate have a higher risk of unwanted pregnancies, self-induced abortion, obstetric complications, and are five times more likely to meet criteria for postpartum depression compared to the general population of host countries [4, 6–8]. These risks have significant consequences for the physical, psychological, and social health of women and their families, including increased risk of maternal morbidity and mortality [3, 9]. In addition, barriers relating to language, acculturation, gender-norms, or religious beliefs may decrease the ability or willingness to seek care in their new home country [10].

To increase refugee women’s reproductive health awareness and improve access to information that influences their sexual and reproductive health (SRH), and SRH care utilization, the Refugee Reproductive Health Network (ReproNet) developed and offered online and in-person reproductive health literacy (RHL) trainings. The training sessions involved videos and online interactive tools in Dari, Pashto, and Arabic. In Fall 2023, in coordination with the Sacramento Public Library and the Muslim American Society-Social Services Foundation (MAS-SSF), ReproNet offered 12 three-session RHL series to Arab and Afghan refugees from Sacramento County, as well as other California counties. The training sessions covered three main topics that had been chosen by refugee women: cervical cancer, family planning, and maternal health/postpartum care [11]. One challenge for the evaluation of these sessions was the identification of validated scales that measure the impact of the training on health literacy.

**Fig. 1:**
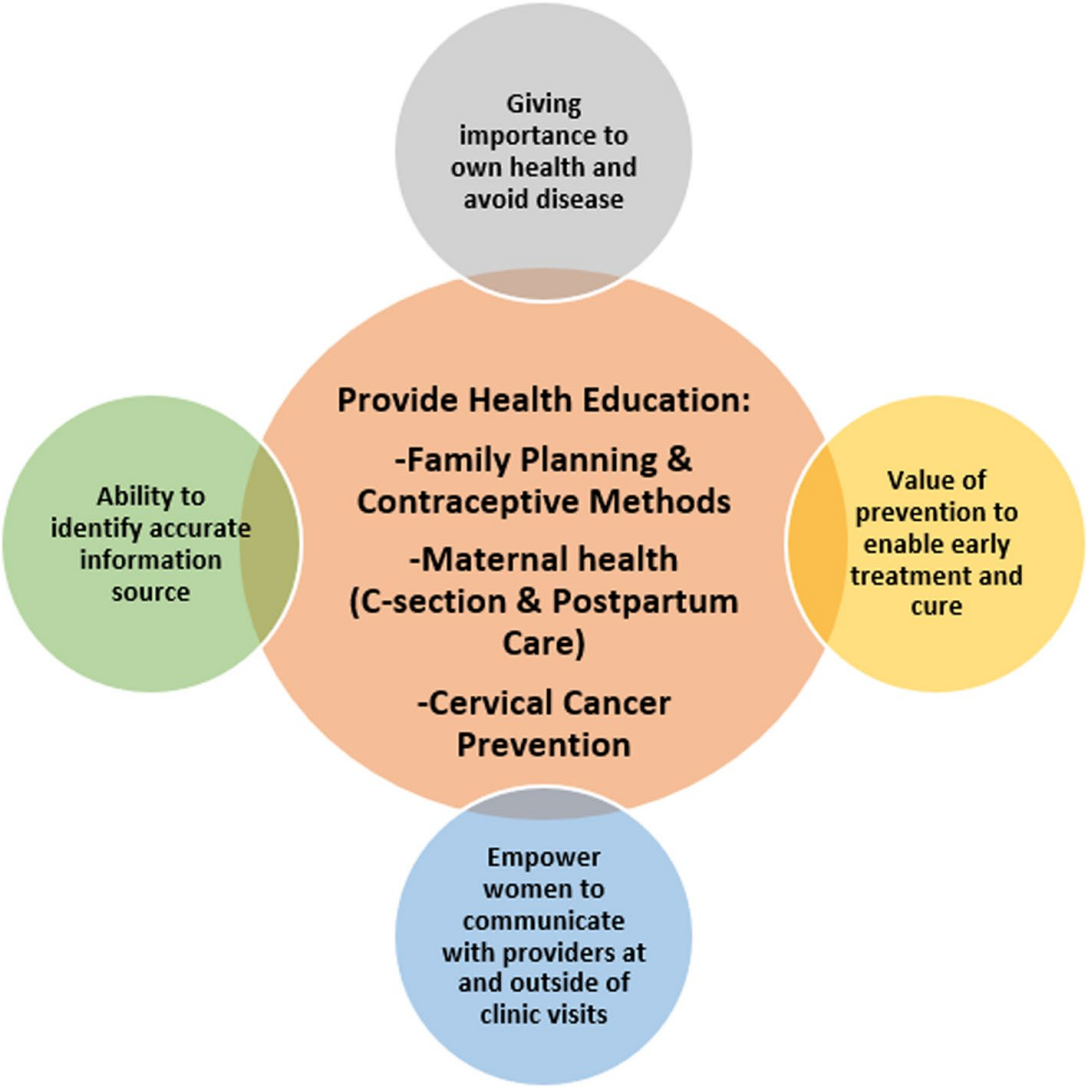
Reproductive health literacy framework

The training format focuses on health literacy as defined in Healthy People 2030 which defined health literacy as “the degree to which individuals have the ability to find, understand, and use information and services to inform health-related decisions and actions for themselves and others” [12]. The respective health content is discussed in the context of strengthening women’s ability to communicate with their medical providers, identify accurate health information sources, and understand the value of preventative care to enable early treatment, and emphasize the importance of their own health and disease prevention (Fig. 1).

Using the Health People 2030 definition of health literacy and the health literacy framework from Fig. 1, this study aimed to examine the existing health literacy tools and develop a culturally and linguistically appropriate instrument that measures reproductive health literacy among refugee women.

## Methods

To develop and validate a reproductive health literacy scale for refugee women, we used the following steps: identification of the needed domains and items, and assessment of content validity, face validity, and inter-item reliability [13].

### Identification of the domains and items

We conducted a literature review of existing reproductive health literacy scales that met the Healthy People 2030 definition to identify the scales that measured the following domains of health literacy: general health literacy, digital health literacy, and reproductive health literacy [12].

Search criteria for scales in PubMed and the Health Literacy Tool Shed included: (a) addressed health literacy according to the Healthy People 2030 definition, (b) covered the area targeted in the RHL trainings and (c) were validated in diverse cultural and linguistic groups. In PubMed, keywords included “health literacy,” “reproductive health,” “scales OR questionnaires,” “digital health OR electronic health OR eHealth”, and “refugee.” In the Health Literacy Tool Shed, the following filters were applied: “health literacy,” “digital health,” “reproductive health,” and “cancer” [14]. The identified scales were evaluated by the research team and ReproNet content experts (international medical graduates) according to the following criteria:

- addresses the content topics of the RHL Training (cervical cancer, family planning, maternal health/postpartum care)
- is aligned with the Healthy People 2030 health literacy definition to assess an individual’s ability to find, understand, and use health information
- reports robust parametrics
- has been piloted with low-income, multicultural populations. Of particular interest were scales that had been tested in Arabic, Dari, and/or Pashto.

Upon review of the scales, we selected the following scales.

### *General health literacy*

For the domain of general health literacy, we selected the European Health Literacy Survey Questionnaire 6 (HLS-EU-Q6). The HLS-EU-Q6 is a shortened version of the European Health Literacy Survey Questionnaire 47 (HLS-EU-Q47) that has been widely used in a variety of countries and was translated and validated in several languages including Persian, Slovenian, Bulgarian, Dutch, German, Greek, Polish, Spanish, Indonesian, Kazakh, Russian, Malay, Myanmar/Burmese, Mandarin, and Vietnamese [15–18]. The HLS-EU-Q6 correlates strongly with the 47- question version (0.896) and is a reliable (*α* = 0.803) measure of health literacy [19]. The 16-question version of the questionnaire (HLS-EU-Q16) has also been administered in Dari, Pashto, and Arabic [20, 21]. We opted to use the Q6 because it could be completed in a shorter duration, while still giving us an accurate measure of general health literacy.

### *Digital health literacy*

For the domain of digital health literacy, we identified the e-Health Literacy Scale (eHEALS). The eHEALS scale assesses the ability to find, understand and use *electronic* health information. Psychometric testing indicated an alpha coefficient for the 8-item scale was 0.88 and factor loadings for each item ranged from 0.60 to 0.84 [22]. This scale has been translated and validated in Arabic while still maintaining strong parametric (*α* = 0.92), and it has been used in Arabic with migrant populations from Syria and Iraq [20].

### *Reproductive health literacy*

The search for scales measuring reproductive health literacy domains provided mixed results. To assess cervical cancer health literacy, we found the 24-item Cervical Cancer Literacy Assessment Tool (C-CLAT) [23]. This tool has a strong factor loading of each item and has been validated in Arabic [23]. For maternal health/postpartum care, we selected five items from a postpartum health literacy scale that was developed and piloted with Arabic immigrants in Southern California (questions 17, 20, 21, 22, 24) [24]. The scale that most closely measured family planning and sexual health literacy was the 40-item Iranian Adult Sexual Health Literacy Assessment Standard Questionnaire (SHELA) [25]. We used two items question 5, “I can obtain information on various methods of pregnancy prevention from various sources” and question 30, “As soon as I realize a sexual problem or disorder, I can find out where or to whom I should go” from this questionnaire [25]. We developed item 23 in our survey, “I know when symptoms after giving birth are so severe that I should see a doctor,” to assess an additional aspect of postpartum health that was not covered in the previous scales.

In order to assess the impact of reproductive health knowledge on changes in health literacy from ReproNet training sessions, we included 3–4 questions per session topic, including questions about cervical cancer, family planning, and maternal health/postpartum care.

### Scale formatting

In the next step we reviewed the response options of the scale. Formatting of responses for HLS-EU-Q6 was kept the same as it was when it was validated, offering the choices “very difficult (=1),” “difficult (=2),” “easy (=3),” and “very easy (=4).” Response options for eHEALS were the same as they were in the study with Arabic speaking migrants, including the choices “strongly disagree (=1),” “disagree (=2),” “agree (=3),” and “strongly agree (=4)” [20]. We deleted the option “don’t know” that was used in the original verbal interview. A 4-point Likert scale was used for the remaining reproductive health literacy questions.

A total of 30 survey items were finalized in English and then translated into Dari, Arabic and Pashto by bilingual ReproNet scholars including trained medical interpreters. In case items were already translated and validated in Arabic or Dari, bilingual and bicultural subject matter experts reviewed the translation for understandability and appropriate use of medical terms. The complete survey was then piloted with bilingual ReproNet volunteers and refugee women for understandability and accuracy.

### Content validity

Content validity of questions was established by review of 11 ReproNet steering committee members, which represent refugee community members, social service and health providers serving refugee communities, refugee health scholars, and subject matter experts on medical accuracy and cultural appropriateness. The surveys were shared and commented on in group e-mails and consensus on the final wording was obtained at steering committee meetings.

### Face validity

Face validity was conducted by having refugee women from the community evaluate the items. For each translated version of the scale, one woman who was comfortable reading and writing in that language and English reviewed the scale and was asked to judge the items based on understandability and appropriateness. Items that everyone agreed to be culturally appropriate, sensitive, and easy to understand were kept. Based on feedback from the ReproNet subject matter experts and community members, we modified several questions on the reproductive health literacy scale to assess a person’s ability to understand and apply knowledge. Question 1 from C-CLAT was modified from “Cervical cancer is preventable” to “I know what I can do to prevent cervical cancer”. Question 10 from C-CLAT was modified from “When detected early, cervical cancer can be cured” to “I understand what can be done if I have an abnormal cervical cancer test.” We originally planned to use subscales from the SHELA. However, upon review by the subject matter experts the wording of the Farsi/Dari items was very complicated and not easily understood in translations to English, Pashto, or Arabic and we chose only two items as described above and designed one new item.

### Survey administration

This study was approved by the University of California, Irvine IRB board. Surveys were administered at training sessions in-person and online. In-person sessions were held at Sacramento Public Library branches. Participants were given a hardcopy of the pre-test survey including demographics questions. Those who were preliterate received help from a bilingual ReproNet volunteer to complete the survey. After the session, survey responses were entered into REDCap by ReproNet research staff. Online sessions were offered via Zoom to pre-registered attendees. Participants received a link to the pre-test REDCap survey which they completed at home, and they were able to request help in completing the survey if needed. For their time, in-person training participants received $20 when they completed the pre-test and online participants $30 gift card if they completed pre- and post-tests.

### Statistical analysis

All statistical analyses were performed using SAS 9.4 (SAS Institute, Cary, NC). Statistical significance was set at *p* < 0.05, using two-tailed tests. We used ANOVA to compare the difference in total mean for each domain of the survey.

## Results

### Participants

Between September 2023 and March 2024, ReproNet offered two series of three-session RHL trainings (in-person and on-line) in Dari, Arabic, and Pashto, respectively. A total of 184 participants (67 Dari, 53 Arabic, and 64 Pashto speakers) completed the pre-test survey.

Arab and Afghan participants exhibited distinct demographic and educational characteristics. Arab speakers were less likely to be married (54.7%) compared to Dari (59.7%) and Pashto (78.1%) speaking participants and more likely to be educated beyond high school (43.4%) compared to Dari (29.9%) and Pashto-speaking participants (0%). While 15.1% of Arabic participants reported not to be able to read and write in English, the proportion was much higher for Dari (32.8%) and Pashto (46.9%) speakers. Nearly all of Dari (91%) and Arabic-speaking (98.1%) participants reported to be able to read and write in Dari and Arabic respectively; whereas only half (53.1%) of Pashto speaking participants were able read and write in Pashto (Table 1).

**Table 1:** Demographics of Arabic, Dari, and Pashto participants including age, age at immigration, number of children, marital status, ability to read/write in English, ability to read/write in preferred language, education level, country of birth, race/ethnicity, and insurance status

	Total *n* = 184	Arabic *n* = 53	Dari *n* = 67	Pashto *n* = 64
*Current age (range 18–64 years)*				
Mean (SD)	35.4 (11.1)	36.3 (12.2)	33.9 (11.3)	36.3 (9.9)
*Age at immigration*				
Mean (SD)	31.3 (13.0)	30.4 (13.4)	30.2 (13.4)	33.1 (12.4)
*# of children (range 0–9 children)*				
Mean (SD)	3.2 (2.5)	2.8 (2.2)	2.8 (2.6)	4.0 (2.5)
*Marital status (n, %)*				
Married/domestic partnership	119 (64.7)	29 (54.7)	40 (59.7)	50 (78.1)
Single, widowed, divorced	42 (22.8)	17 (32.1)	17 (25.4)	8 (12.5)
*Read/write in English (n, %)*				
Can only read, only write, or can do both	120 (65.1)	45 (85.0)	44 (65.7)	31 (48.5)
Neither	60 (32.6)	8 (15.1)	22 (32.8)	30 (46.9)
*Read/write in preferred language (n, %)*				
Can only read, only write, or can do both	152 (82.7)	52 (98.1)	61 (91.0)	39 (61.0)
Neither	29 (15.8)	1 (1.9)	6 (9.0)	22 (34.4)
*Education level (n, %)*				
Up to high school	136 (73.9)	29 (54.7)	46 (68.7)	61 (95.3)
Some college or above	43 (23.4)	23 (43.4)	20 (29.9)	0 (0.0)
*Country of birth (n, %)*				
Afghanistan	116 (62.4)	0 (0.0)	62 (92.5)	54 (81.8)
Syria	33 (17.7)	33 (62.3)	0 (0.0)	0 (0.0)
United States	5 (2.7)	5 (9.4)	0 (0.0)	0 (0.0)
Other (including Iraq, Egypt, Palestine, etc.)	8 (4.3)	6 (11.4)	2 (3.0)	0 (0.0)
*Race/ethnicity (n, %)*				
Asian	116 (63.0)	9 (17.0)	54 (80.6)	53 (82.8)
Middle Eastern/North African	33 (17.9)	31 (58.5)	2 (3.0)	0 (0.0)
White	13 (7.1)	6 (11.3)	3 (4.5)	4 (6.3)
Black/other race	22 (11.9)	7 (13.2)	8 (11.9)	7 (10.9)
*Insurance (n, %)*				
Medicaid/Medi-Cal	155 (84.3)	40 (75.5)	53 (79.1)	62 (96.9)
None	6 (3.2)	2 (3.8)	3 (4.5)	1 (1.6)
Other	18 (9.7)	11 (20.7)	7 (10.5)	0 (0.0)

### Inter-item reliability analysis

We calculated the mean and standard deviations for each scale item as well as the total mean for each domain of the survey for each language group (Table 2). The mean scores for individual items ranged from 2.24 to 2.87 on a 4-point Likert scale. The differences in the mean score obtained between each language group was statistically significant for the domain of general health literacy (*p* = 0.0002) but not statistically significant for digital health literacy (*p* = 0.8684) or RHL (*p* = 0.9975).

**Table 2:** Mean and standard deviation scores of Scale Questions of Total, Arabic, Dari, and Pashto participants

		Total		Arabic		Dari		Pashto		
		Mean	SD	Mean	SD	Mean	SD	Mean	SD	
*HLS-EU-Q6: On a scale from very easy to very difficult, how easy would you say it is to…*										
1	Judge when you may need to get a second opinion from another doctor	2.54	0.89	2.53	0.67	2.23	0.84	2.88	0.99	
2	Use information the doctor gives you to make decisions about your illness	2.72	0.84	2.7	0.64	2.41	0.89	3.08	0.8	
3	Find information on how to manage mental health problems like stress or depression	2.49	0.91	2.47	0.64	2.23	1.02	2.79	0.9	
4	Judge if the information on health risks in the media is reliable	2.51	0.9	2.49	0.67	2.26	0.96	2.79	0.94	
5	Find out about activities that are good for your mental well-being	2.72	0.97	2.6	0.75	2.65	1.09	2.89	1.0	
6	Understand information in the media on how to get healthier	2.8	0.85	2.63	0.6	2.83	0.95	2.92	0.9	
Total HLS-EU-Q6		2.63	0.65	2.57	0.46	2.43	0.67	2.89	0.7	0.0002
*eHEALS*										
7	I know how to find helpful health resources on the Internet	2.79	0.85	2.89	0.64	2.85	0.9	2.63	0.94	
8	I feel confident in using information from the Internet to make health decisions	2.74	0.8	2.62	0.66	2.89	0.84	2.68	0.85	
9	I know where to find helpful health resources on the Internet	2.64	0.83	2.51	0.58	2.71	0.95	2.69	0.89	
10	I know how to use the Internet to answer my health questions	2.71	0.81	2.73	0.74	2.64	0.88	2.75	0.81	
11	I know what health resources are available on the Internet	2.61	0.82	2.57	0.67	2.58	0.94	2.69	0.83	
12	I can tell high quality from low quality health resources on the Internet	2.54	0.8	2.53	0.72	2.48	0.84	2.6	0.83	
13	I know how to use the health information I find on the Internet to help me	2.66	0.8	2.6	0.6	2.72	0.86	2.66	0.89	
14	I have the skills I need to evaluate the health resources I find on the Internet	2.61	0.82	2.57	0.72	2.65	0.86	2.61	0.86	
Total eHEALS		2.65	0.67	2.63	0.5	2.69	0.69	2.64	0.77	0.8684
*Reproductive health (RH) literacy*										
15	I know what I can do to prevent cervical cancer	2.24	1.03	2.25	0.76	2.02	1.16	2.48	1.05	
16	I understand what can be done if I have an abnormal cervical cancer test	2.33	1.03	2.26	0.76	2.27	1.22	2.46	1.01	
17	I understand how the reproductive system works	2.41	1.05	2.51	0.78	2.32	1.25	2.41	1.04	
18	As soon as I realize a sexual problem or disorder, I can find out where or to whom I should go	2.63	1.04	2.55	0.8	2.63	1.24	2.69	0.99	
19	I can obtain information on various methods of pregnancy prevention from various sources	2.69	1.03	2.68	0.75	2.68	1.24	2.71	1.02	
20	I know how to get information about healthy nutrition before getting pregnant	2.68	1.04	2.68	0.73	2.74	1.24	2.61	1.04	
21	I know when and where to go for the necessary tests or examinations when I am pregnant	2.87	0.99	2.98	0.57	2.89	1.23	2.73	0.99	
22	I can find out how to schedule check-ups for myself or a friend after giving birth	2.63	1.05	2.75	0.72	2.59	1.29	2.57	1.01	
23	I know when symptoms after giving birth are so severe that I should see a doctor	2.8	0.99	2.6	0.72	3.0	1.16	2.75	0.98	
24	I know how to identify symptoms of postpartum depression	2.6	1.05	2.53	0.78	2.6	1.27	2.67	1.0	
Total RH literacy		2.58	0.81	2.58	0.55	2.58	0.96	2.59	0.83	0.9975

We tested each scale for inter-item reliability. The inter-item reliability scores for all participants and by language group (Dari, Arabic, and Pashto) for the three domains of the survey. For general health literacy, digital health literacy, and reproductive health literacy, alpha coefficients were all greater than 0.7, indicating a good inter-item reliability (Table 3). This also stood true across each language group. The alpha coefficient calculated for eHEALS was 0.91, which was very similar to the (*α*) of 0.88 calculated in the original scale [22]. The total scores of the three health literacy domains were highly correlated with Pearson Correlation ranging from 0.21 to 0.50.

**Table 3:** Alpha coefficients of completed Total, Arabic, Dari, and Pashto RHL training pre-tests

Scale	Total *n* = 184	Arabic *n* = 53	Dari *n* = 67	Pashto *n* = 64
General health literacy (EU-HLS-6)	0.8218	0.7716	0.7638	0.8665
Digital health literacy (e-Heals)	0.9217	0.8954	0.9069	0.9512
Reproductive health literacy	0.9302	0.9027	0.9291	0.9466

## Discussion

Refugee service providers are increasingly aware of the need to address refugee communities’ reproductive and sexual health. Existing health education programs tend to focus on knowledge acquisition, rather than enabling women to make informed health decisions and advocate for their health.

In order to evaluate an innovative reproductive health literacy training for refugee women, we developed a reproductive health literacy scale that assesses training impact on health literacy, digital health literacy, and reproductive health literacy based on existing validated scales wherever possible. We could show that the domains of general health literacy (HLS-EU-Q6), digital literacy (eHEALS), and reproductive health literacy can be combined in one survey instrument that can be used in on-line and in-person group sessions. The administration of the tool and robust parametrics to three refugee groups with diverse demographic and linguistic characteristics shows the potential for this scale to be adapted to additional languages.

The reproductive health literacy scale included items that measure reproductive health topics addressed in our reproductive health training. The means for items from HLS-EU-Q6 for Dari speaking participants resembled those seen in the literature, ranging from 2.13 to 2.46, with our values ranging from 2.49 to 2.80 (Table 2) [21]. The range of means for all items suggested that participants understood the response options and felt comfortable with the scale. The mean health literacy scores of Arab speaking training participants were higher than those of Dari speaking training participants, consistent with Arab immigrants’ higher education level, longer stay in the US and greater familiarity with digital media. While there were differences in the mean scores across the language groups in general health literacy, participants in all three groups scored similarly in other domains. This discrepancy will be further explored in a future analysis of a larger sample controlling for covariates. The internal consistency of the scales in Dari and Arabic were consistent with the values of the original studies [21].

Other existing scales that have been identified in the literature review covered limited aspects of reproductive health and/or were developed for specific target populations like adolescents [26]. For example, the Iranian Adult Sexual Health Literacy Assessment Standard Questionnaire (SHELA) focused primarily on aspects of sexual health, including STIs, partner safety and contraception [25]. Another survey of reproductive health literacy only asked knowledge-based questions and was not validated [27]. The Sexual and Reproductive Health Literacy questionnaire was validated in adolescents aged 15–19, but it focused mainly on issues of sexual health in people of this age group [26]. We also found the Reproductive Health Literacy Questionnaire for Chinese Unmarried Youth which covered the topics: physiological and psychological development during adolescence, personal health care during adolescence, heterosexual relationship and sexual behavior, prevention and response to sexual harassment and sexual abuse, prevention of AIDS and STDs, and prevention and response to unintended pregnancy [28]. These scales could potentially provide items for topics that were not addressed in our reproductive health literacy scale.

A limitation of this survey is that it does not address all reproductive health domains such as sexual health. We also did not include questions about comfort communicating with providers or behavioral intent to keep the survey length feasible to administer. However, we were able to identify scales with robust reliability for general and domain specific health literacy. Another limitation is that participants with low literacy skills needed help completing the questionnaire due to limited reading/writing ability. This could potentially lead to a social desirability bias. We addressed this through training of the research assistants and volunteers.

## Conclusions

Our reproductive health literacy survey creates a measure to assess the impact of health education initiatives on the ability to assess health information and use it for informed health decisions, complementing qualitative assessments of health education sessions. This has significant utility in the use of measuring effectiveness of health literacy training for research purposes but also for the purposes of health promotion and prevention of disease. Additionally, it can be used to investigate relationships with other domains such as reproductive autonomy. This measure promises to provide the ability to compare the effectiveness of health interventions for newcomers with different cultural and linguistic backgrounds. Future research with this scale will involve investigating the differences in general, digital, and reproductive health literacy among Dari, Pashto, and Arabic speaking refugees alongside ReproNet RHL trainings with pre- and post-test analysis.

## Data Availability

The datasets used and/or analyzed during the current study are available from the corresponding author on reasonable request.

## Abbreviations

RH: Reproductive health
SIV: Special Immigrant Visa
SRH: Sexual and reproductive health
ReproNet: Refugee Reproductive Health Network
RHL: Reproductive health literacy
MAS-SSF: Muslim American Society-Social Services Foundation
HLS-EU-Q6: European Health Literacy Survey Questionnaire 6
HLS-EU-Q47: European Health Literacy Survey Questionnaire 47
HLS-EU-Q16: European Health Literacy Survey Questionnaire 16
eHEALS: E-Health Literacy Scale
C-CLAT: Cervical Cancer Literacy Assessment Tool
SHELA: Iranian Adult Sexual Health Literacy Assessment Standard Questionnaire
